# Enzyme Method-Based Microfluidic Chip for the Rapid Detection of Copper Ions

**DOI:** 10.3390/mi12111380

**Published:** 2021-11-10

**Authors:** Binfeng Yin, Xinhua Wan, Changcheng Qian, A. S. M. Muhtasim Fuad Sohan, Teng Zhou, Wenkai Yue

**Affiliations:** 1 School of Mechanical Engineering, Yangzhou University, Yangzhou 225127, China; usawwns@163.com (X.W.); qcc825690832@foxmail.com (C.Q.); hyperdrive4u@icloud.com (A.S.M.M.F.S.); wkyue0905@foxmail.com (W.Y.); 2 Mechanical and Electrical Engineering College, Hainan University, Haikou 570228, China; zhouteng@hainanu.edu.cn

**Keywords:** biological logic gate, Cu^2+^ detection, microfluidic chip

## Abstract

Metal ions in high concentrations can pollute the marine environment. Human activities and industrial pollution are the causes of Cu^2+^ contamination. Here, we report our discovery of an enzyme method-based microfluidic that can be used to rapidly detect Cu^2+^ in seawater. In this method, Cu^2+^ is reduced to Cu^+^ to inhibit horseradish peroxidase (HRP) activity, which then results in the color distortion of the reaction solution. The chip provides both naked eye and spectrophotometer modalities. Cu^2+^ concentrations have an ideal linear relationship, with absorbance values ranging from 3.91 nM to 256 μM. The proposed enzyme method-based microfluidic chip detects Cu^2+^ with a limit of detection (LOD) of 0.87 nM. Other common metal ions do not affect the operation of the chip. The successful detection of Cu^2+^ was achieved using three real seawater samples, verifying the ability of the chip in practical applications. Furthermore, the chip realizes the functions of two AND gates in series and has potential practical implementations in biochemical detection and biological computing.

## 1. Introduction

Marine environmental pollution is an increasingly acute global problem [1,2]. Trace metals are essential components of biogeochemistry; however, high concentrations of metals can cause persistent harm to the marine environment [3,4]. Copper is a common micronutrient. Copper ions found in seawater mainly exist in the form of Cu^2+^ in the concentration of fM-pM [5]. Concentrations vary depending on the location, temperature, acidity, salinity, and depth of the seawater [6]. The concentration of copper ions in the ocean can rise to μM as a consequence of human activities and industrial pollution. High concentrations of copper ions are toxic, particularly to the metabolisms of marine organisms [7]; for example, high levels of copper ions can interfere with the fertilization of sea urchins and negatively impact algae survival [8,9]. For humans, excess copper ions can lead to diseases such as anemia and Alzheimer’s disease [10,11,12]. According to the China Sea Water Quality Standard, the concentration of copper in seawater that comes into contact with human beings should be lower than 0.16 μM. Therefore, the rapid detection of copper ion concentration in seawater has practical significance for marine environment protection.

In recent years, researchers have developed numerous Cu^2+^ detection methods, including fluorescence, colorimetry, and electrochemical sensors [13,14,15,16,17,18]. Tian et al. reported the successful use of a fluorescence sensor for rapidly detecting Cu^2+^ [19]. The mechanism of the sensor uses the photoinduced electron transfer effect on Cu^2+^ in ultrathin graphite carbon nitride nanosheets, which leads to the fluorescence extinction of the sensor. Whilst this method is extremely sensitive, it relies on large instruments, and the preparation of nanosheets is complex. Colorimetry provides a convenient method for the detection of Cu^2+^. Ye et al. described the use of rhodamine B and 5-ferrocenyl-1,3,4-thia-diazole to create a rhodamine probe [20]. Cu^2+^ causes the ring-opening of rhodamine spiro lactam, turning the colorless solution pink. This method has a noticeable readout effect but low sensitivity. Developing a detection method for Cu^2+^ in seawater offers the potential for convenient readouts, high sensitivity, and a wide detection range, making this concept worthy of research.

Microfluidic chips are capable of completely integrating biochemical reactions [21,22]. High throughput, small size, low cost, and support for personalized design are features of microfluidic chips [23,24]. In recent years, due to the state-of-the-art attributes of microfluidic platforms, they have become mainstream in environmental detection. Microfluidic chip platforms enable the reliable detection of markers using nanoparticles, fluorescent probes, and color reactions [25,26]. Idros et al. created a multiplex detection technique for heavy metals [27]. Their approach integrated a microfluidic chip to detect heavy metals in water. We believe that it is feasible to develop a Cu^2+^ detection method based on a microfluidic chip.

Herein, we explored the potential of an enzyme method-based microfluidic chip for rapidly detecting Cu^2+^ in seawater. The microfluidic chip produces Cu^+^ by reducing Cu^2+^ with sodium ascorbate, inhibiting horseradish peroxidase (HRP) activity. Tetramethylbenzidine (TMB) acts as the start key and reacts with HRP to form a blue solution, while HCl acts as the end key to stop the reaction and form a yellow solution. The signal of 3.91–256,000 nM Cu^2+^ can be read out by both the naked eye and spectrophotometry. The enzyme method-based microfluidic chip provides an expeditious detection of Cu^2+^ in seawater and a potential direction for the development and future study of biological logic gates and biological computers.

## 2. Materials and Methods

### 2.1. Materials and Instruments

HRP was purchased from Solarbio Science & Technology Co., Ltd. (Beijing, China). TMB solution was obtained from Makewonderbio Co., Ltd. (Beijing, China). Phosphate Buffered Saline (PBS) tablets were purchased from Amresco (Pittsburgh, PA, USA) to prepare 0.01 mol/L PBS solution (pH 7.4). PBS solution was used to dilute HRP. Other chemicals were analytical grade and diluted in distilled water with a salinity of 3.5% to simulate a seawater environment. The microfluidic chip was made from Polydimethylsiloxane (PDMS) and a curing agent (Sylgard 184) purchased from Dow Corning Inc. (Rochester, NY, USA). Molds of the microfluidic chip were made from Lasty-R resin from SHINING 3D Technology Co., Ltd. (Hangzhou, China).

A Lite 600HD 3D printer from SHINING 3D Technology Co., Ltd. (Yangzhou, China) was used to manufacture the molds. LICHEN-BX Co., Ltd. (Shanghai, China) provided a DZF-6020A vacuum drying oven and a 202-00T electric thermostatic drying. A PT-10s Plasma Cleaner was purchased from SANHOPTT Co., Ltd. (Shenzhen, China). INESA Analytical Instrument Co., Ltd. (Shanghai, China) produced the L5S UV/VIS Spectrophotometer.

### 2.2. Design and Fabrication of the Microfluidic Chip

The microfluidic chip comprises double PDMS layers and measures 58 mm in length, 24 mm in width, and 8 mm in thickness in total. The function layer stores reagents and implements the operation of the enzyme method-based microfluidic chip. It contains three input reservoirs and two control reservoirs. Microchannels both 400 μm wide and deep connect all reservoirs. We reduced the thickness above the reaction reservoir to prevent excessive PDMS from affecting the color signal readout when performed by the naked eye. Drilling holes in each reservoir facilitates the injection of reagents and equalizes pressure when positive pressure increases. The readout layer consists of a reaction reservoir. The reaction takes place in the reaction reservoir, from which readouts can be observed.

**Figure 1:**
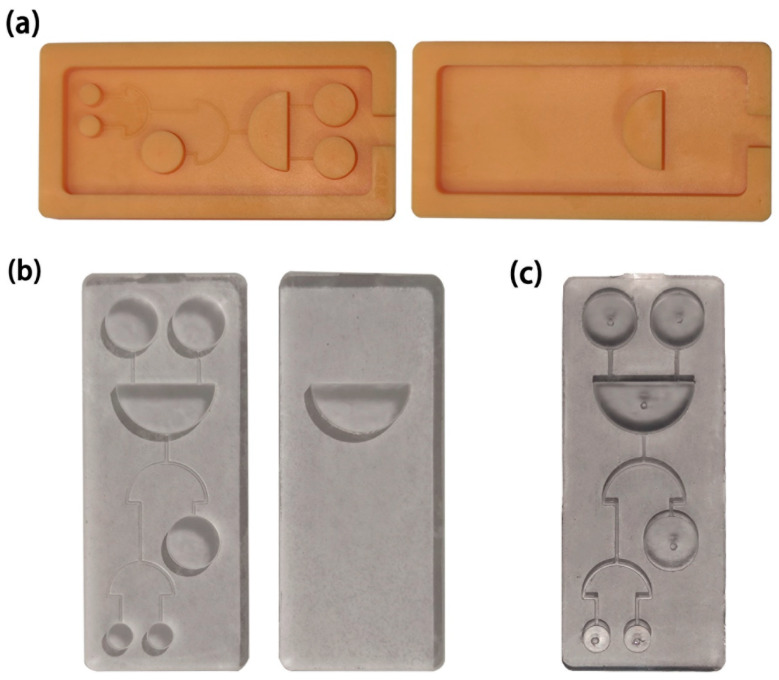
Photos of the manufacturing stages of the microfluidic chip: (**a**) molds of the microfluidic chip. (**b**) The function layer and readout layer of the microfluidic chip. (**c**) The assembled microfluidic chip.

As an emerging manufacturing technology, 3D printing has the characteristics of individuation and high precision. It has been widely used in miniaturized mechanical and electromechanical elements to efficiently manufacture microdevices in recent years [28,29]. We used a high-precision desktop 3D printer to make the molds of the microfluidic chip (Figure 1a). After cleaning, curing, and polishing the molds, gloss oil was sprayed on the molds to facilitate curing and stripping, and to improve the surface roughness of the double PDMS layers. We then mixed the PDMS and curing agents with a mass ratio of 9.5:1 and removed the bubbles in a vacuum drying oven, before pouring them into molds. We stored the molds at 85 °C for 60 min to cure the PDMS and then demolded the double PDMS layers (Figure 1b). The plasma cleaner modified the surface of the double PDMS layers and made them bond tightly after pressing (Figure 1c).

### 2.3. Optimization of Reaction Conditions

**Figure 2:**
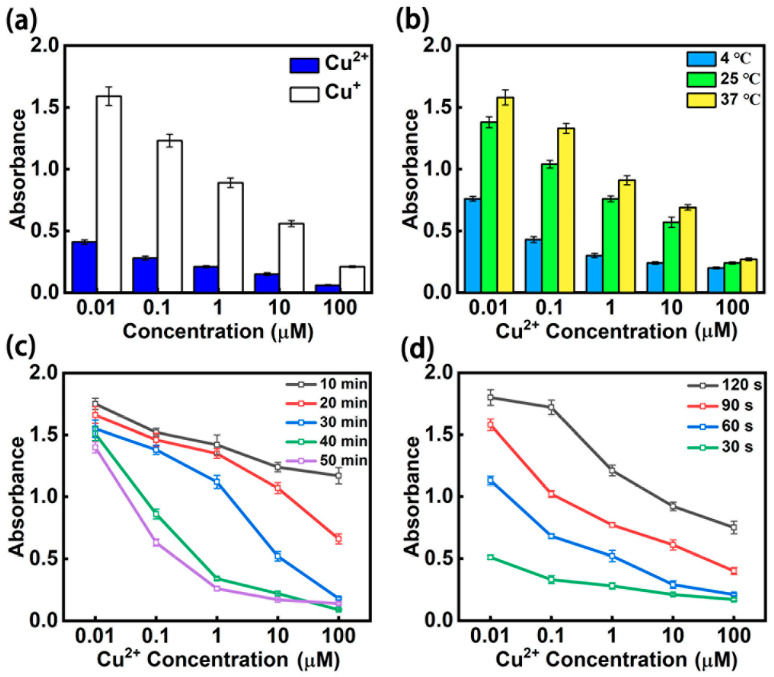
Optimization of operating conditions for the enzyme method-based microfluidic chip: (**a**) inhibition effect of Cu^2+^ and Cu^+^ on HRP activity. (**b**) Effect of temperature on the operation of chip. (**c**) Influence of incubation time on signal intensity. (**d**) Detection results at different readout times.

Sodium ascorbate is a commonly used reducing agent in the sensing stage of biochemical detection. It does not inhibit HRP activity. In addition, Cu^2+^ and Cu^+^ generated by a reduction reaction may affect HRP activity. In order to explore the factors of the inhibition of HRP activity, we reacted different concentrations of Cu^2+^ and Cu^+^ with 12.5 ng/mL HRP, respectively (Figure 2a). The results showed that Cu^2+^ had a slight inhibitory effect on HRP activity, while Cu^+^ had a more substantial effect. Therefore, we needed to completely reduce Cu^2+^ to Cu^+^ in the chip to attain a more obvious signal.

The reaction temperature was an essential factor, as it could affect results and therefore the practical application of the chip. To investigate the optimal application temperature of the chip, we conducted experiments at 4 °C, 25 °C, and 37 °C, respectively (Figure 2b). Although we obtained a good signal intensity strength at 25 °C, the strongest signal intensity was at 37 °C, which is consistent with the optimal reaction temperature of HRP. The concentration of Cu^2+^ in normal seawater is extremely low, so we needed to obtain a higher sensitivity to meet the detection requirements. In the end, we chose 37 °C as the reaction temperature. Using a miniature thermostat did not cause inconvenience in the actual detection.

Incubation time was the most time-consuming part of the chip’s operation. To obtain a clear signal in a short time, we explored the influence of different incubation times on signal intensity (Figure 2c). HRP activity was not completely inhibited when the incubation time was too short, but even a low concentration of Cu^2+^ could significantly inhibit HRP activity when incubation time was too long. Choosing 30 min as the incubation time ensured evident signal intensity at different concentrations of Cu^2+^.

HRP and TMB react quickly, so readout time has a significant effect on signal intensity. We investigated the signal intensity obtained at different readout times (Figure 2d). The short readout time did not produce a clear signal, while the long readout time caused the color to darken continuously. For a microfluidic chip, 90 s is the best readout time. Since microfluidic chips can be automatically controlled using portable instruments, it is not difficult to accurately control the readout time.

## 3. Results and Discussion

### 3.1. Principle

The HRP-TMB color reaction is a well-known technique in biochemical detection. TMB participates as a hydrogen donor in the HRP-catalyzed H_2_O_2_ reduction process. TMB is oxidized during the process, resulting in the formation of diimine, which turns the solution blue. This color change produces absorption peaks at 371 nm and 652 nm [30]. The addition of acid can stop the reaction and turn the solution yellow. It has an absorption peak at 450 nm. Both Cu^2+^ and Cu^+^ inhibit HRP activity in the form of Cu^+^. When only Cu^2+^ reacts with HRP, the potential reducibility of protein may cause Cu^2+^ to be reduced. The porphyrin ligand is generally considered the active site of HRP. However, inhibition of HRP activity by Cu^+^ may occur on amino acid residues [31]. The specific sites of inhibition need further study.

### 3.2. Detecting Process

**Figure 3:**
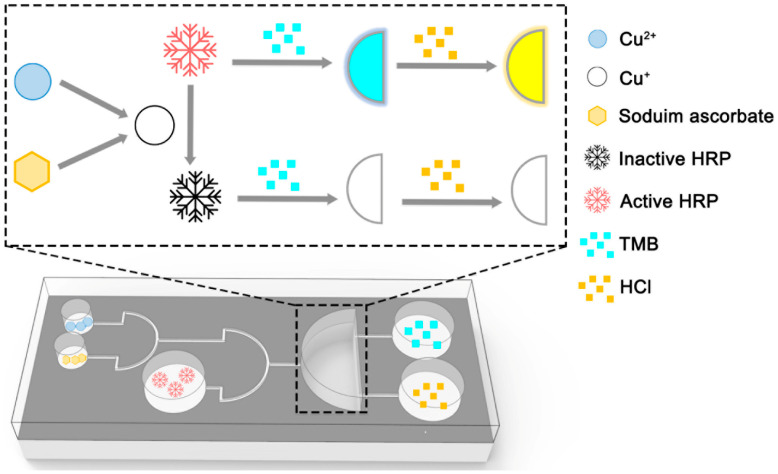
The detection mechanism of the fabricated enzyme method-based microfluidic chip.

We added 5 μL of the test samples with different concentrations of 5 μL 100 μM sodium ascorbate, and 90 μL 12.5 ng/mL HRP into input reservoirs a, b, and c, respectively. Then, 100 μL TMB solution and 50 μL 2 M HCl were stored in control reservoirs a and b. For driving the reagents, a positive pressure was applied. First, we pumped all the reagents from the input reservoirs into the reaction reservoir, then incubated at 37 °C for 30 min. After that, the TMB solution was pumped into the reaction reservoir to react with the HRP. In order to stop the reaction after 90 s (Figure 3), stored HCl from the control reservoir was pumped into the reaction reservoir. Finally, the enzyme method-based microfluidic chip provided two methods for the reading out of signals. Firstly, they could be read with the naked eye. The solution of Cu^2+^ pollution with a standard concentration was prepared into a colorimetric standard in the chip. By comparison with the colorimetric standard, a rough estimate of the pollution of Cu^2+^ could be achieved. Secondly, the solution could be taken from the reaction reservoir and put into a spectrophotometer to detect the absorbance of the solution at 450 nm. The absorbance could then be used to accurately calculate the concentration of Cu^2+^.

### 3.3. Detecting Performance

**Figure 4:**
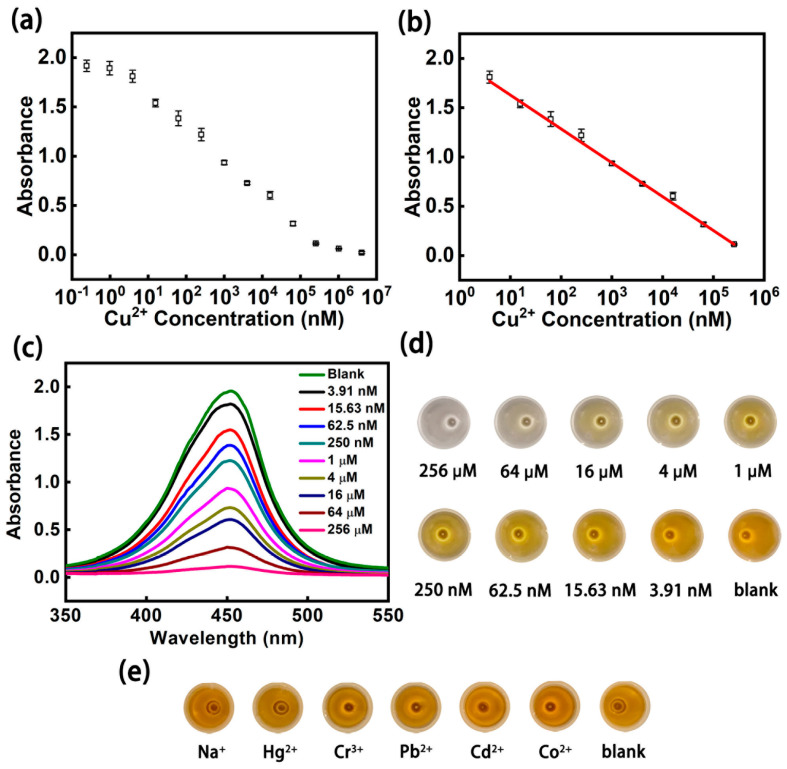
The detection performance of the biological logic gates: (**a**) detecting results of Cu^2+^ in the range of 0.24 nM–4096 μM. (**b**) Linear curve of Cu^2+^ at 3.91 nm–256 μM. (**c**) Uv-vis spectra of Cu^2+^ at different concentrations in the linear range. (**d**) Color results of different concentrations of Cu^2+^ in the linear range. (**e**) Color results of other metal irons.

We used linear range, limit of detection (LOD), selectivity, repeatability, and storage stability to evaluate the chip’s detection performance. The detection of 0.24 nM–4096 μM Cu^2+^ demonstrated an excellent linear relationship between Cu^2+^ levels and the absorbance value at the concentration of 3.91 nM–256 μM ($Y=-0.34374\;\cdot \;X+1.97302,{\;R}^{2}=0.998$). The linear range shows that the chip can detect the concentration of Cu^2+^ in seawater with different pollution levels (Figure 4a–d). LOD was calculated by dividing the standard deviation of the blank sample by the slope of the linear curve. The LOD of Cu^2+^ for the chip is 0.87 nM. Since seawater contains other metal ions, it was essential to determine whether the presence of other metal ions would affect the detection of Cu^2+^. We used the enzyme method-based microfluidic chip to detect Na^+^, Hg^2+^, Cr^3+^, Pb^2+^, Cd^2+^, and Co^2+^ at 1 μM (Figure 4e). The results show that other metal ions do not inhibit HRP activity and do not affect the detection of Cu^2+^. Repeatability and storage stability are preconditions for the practical application of the enzyme method-based microfluidic chip, which is similar to a biological logic gate. Ten chips were used to detect the solution with a Cu^2+^ concentration of 1 μM. Their coefficient of variation (CV) was 3.45% (Figure S1). The CV of the chips was 4.53% after they were stored at 4 °C for 1–5 days (Figure S2) for detecting 1 μM Cu^2+^ solution. In order to verify the detection ability of the chip, we compared it with colorimetric, fluorescence, and photoelectrochemical methods (Table S1). The results show that the chip has the advantages of low cost, easy operation, wide linear range, and high sensitivity. The detection performance of the chip met the detection demand of Cu^2+^ in seawater.

### 3.4. Detection of Actual Samples

**Figure 5:**
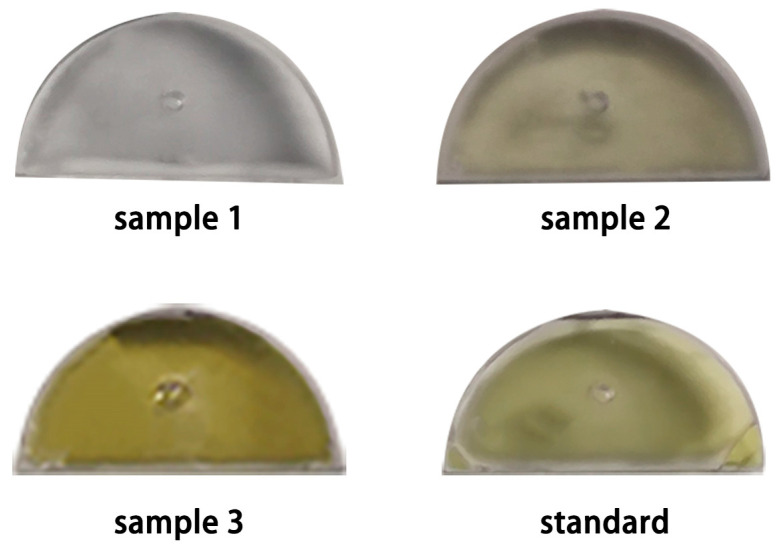
Color result diagrams of actual samples detected by biological logic gates.

To verify the performance of the enzyme method-based microfluidic chip in detecting actual seawater samples, we collected three seawater samples with different levels of pollution (Lianyungang, China). The standard color sample was obtained by detecting the standard solution of 0.16 μM Cu^2+^, which the China Sea Water Quality Standard requires. We performed real-time detection at the sampling site and read out the microfluidic chip’s signal intensity with the naked eye (Figure 5). The color of the solution in the reaction reservoir was compared to the color of the standard sample to determine the pollution level. A sample solution from the chip was tested in a lab setting using a spectrophotometer to acquire precise data. Results of the two readout methods of the chip were compared with those of ICP-OES as a reference (Table 1). The results showed that the naked eye readout could judge pollution and that using the spectrophotometer results could accurately detect Cu^2+^ concentration.

### 3.5. Operation of Microfluidic Chip

**Figure 6:**
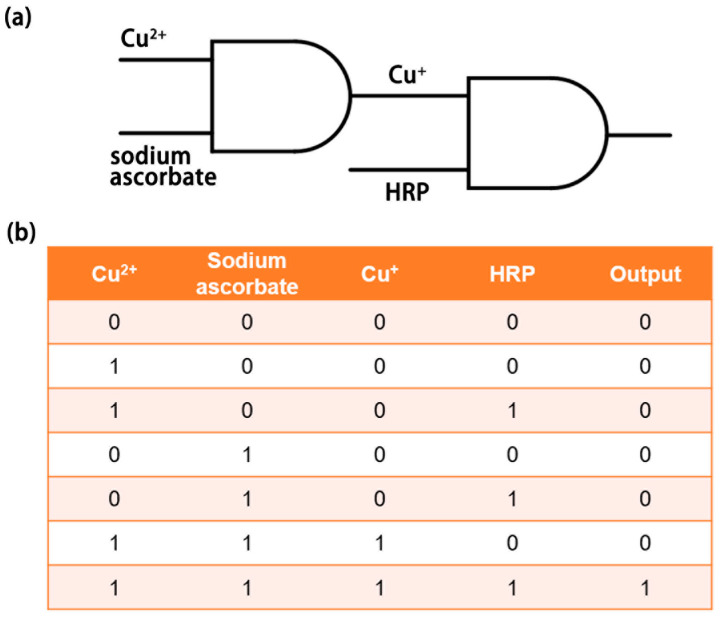
The logical structure of biological logic gates: (**a**) schematic of a biological logic gate. (**b**) Truth table for biological logic gates.

Based on an enzyme method, the microfluidic chip constructs two AND gate structures in series (Figure 6a). Cu^2+^ and sodium ascorbate are inputs of the first AND gate. Cu^+^ produced by reduction reaction is the output of the first AND gate and one of the inputs of the second AND gate. The other input of the second AND gate is HRP. Inhibition of HRP activity is the output of the second AND gate. The reagent is ‘1’ in the presence and ‘0’ in the absence. TMB outputs HRP activity by color as the start key of the enzyme method-based microfluidic chip aspired biological logic gate. HCl acts as the end key of the biological logic gate to terminate the reaction in the reaction reservoir and make the solution present its final color. The output of the whole biological logic gate is ‘0’ for a yellow solution and ‘1’ for a colorless solution. It can be seen from the truth table of the biological logic gate that the final output will be ‘1’ only when the three inputs of Cu^2+^, ascorbic acid, and HRP are ‘1’ at the same time (Figure 6b). The result satisfies the reality of the double AND gate series. Since Cu^2+^ and sodium ascorbate are inexpensive reagents, and unlabeled HRP is common, the cost of biological gates is low. It means that visual biological logic gates have potential applications in biological computers.

## 4. Conclusions

In this work, we developed a biological gate based on a microfluidic chip platform to detect Cu^2+^ in seawater. In the biological logic gate, Cu^2+^ and sodium ascorbate produce Cu^+^. Cu^+^ reacted with HRP amino acid residues to inhibit HRP activity. A color change occurred when the TMB solution was added. Both the naked eye and a spectrophotometer can be used to read out the signal intensity. Biological logic gates had an excellent detection performance, including in the detection of actual seawater samples. The enzyme method-based microfluidic chip provides a convenient and low-cost platform, laying a foundation for promoting biological logic gates. The biological logic gate realizes the function of two AND gates in series and can read the result visually. Biological gates provide a new method for detecting Cu^2+^ in seawater and have a potential for development in the field of biological computers.

## Figures and Tables

**Table 1:** Comparison of actual sample results by different methods.

	Naked Eyes	Spectrophotometer	ICP-OES
Sample 1	polluted	218.74 μM	217.31 μM
Sample 2	polluted	21.29 μM	19.78 μM
Sample 3	unpolluted	14.57 nM	14.31 nM
Location	Sampling site	Lab	Lab

## Supplementary Materials

The following are available online at https://www.mdpi.com/article/10.3390/mi12111380/s1, Figure S1: Reproducibility of the biological logic gates; Figure S2: Storage stability of the biological logic gates; Table S1: Comparison of biological logic gates detection capabilities.
