# Supplementary material for: Enzyme Method-Based Microfluidic Chip for the Rapid Detection of Copper Ions
Source: Micromachines (Basel). 2021 Nov 10;12(11):1380. doi: 10.3390/mi12111380 (PMC8622971; doi:10.3390/mi12111380)
Supplement: Supplementary file 1 [file micromachines-12-01380-s001.zip › micromachines-1436947-supplementary.pdf]

micromachines

MDPI

Supplementary Information

Enzyme Method-Based Microfluidic Chip for the Rapid Detection of Copper Ions

Binfeng Yin[1*], Xinhua Wan[1], Changcheng Qian[1], A. S. M. Muhtasim Fuad Sohan[1], Teng Zhou[2] and Wenkai Yue[1]

[1] School of Mechanical Engineering, Yangzhou University, Yangzhou 225127, China;
[2] Mechanical and Electrical Engineering College, Hainan University, Haikou 570228, China;
* Correspondence: binfengyin@yzu.edu.cn.

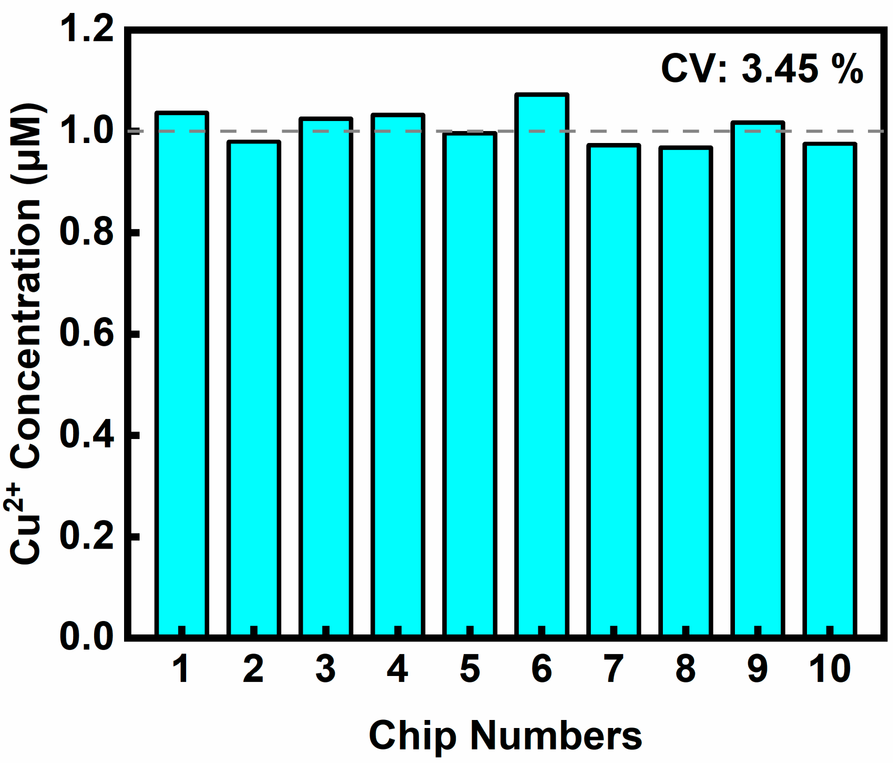

Figure S1. Reproducibility of the biological logic gates. The quantitative results of 1 μM Cu^{2+} in 10 chips.

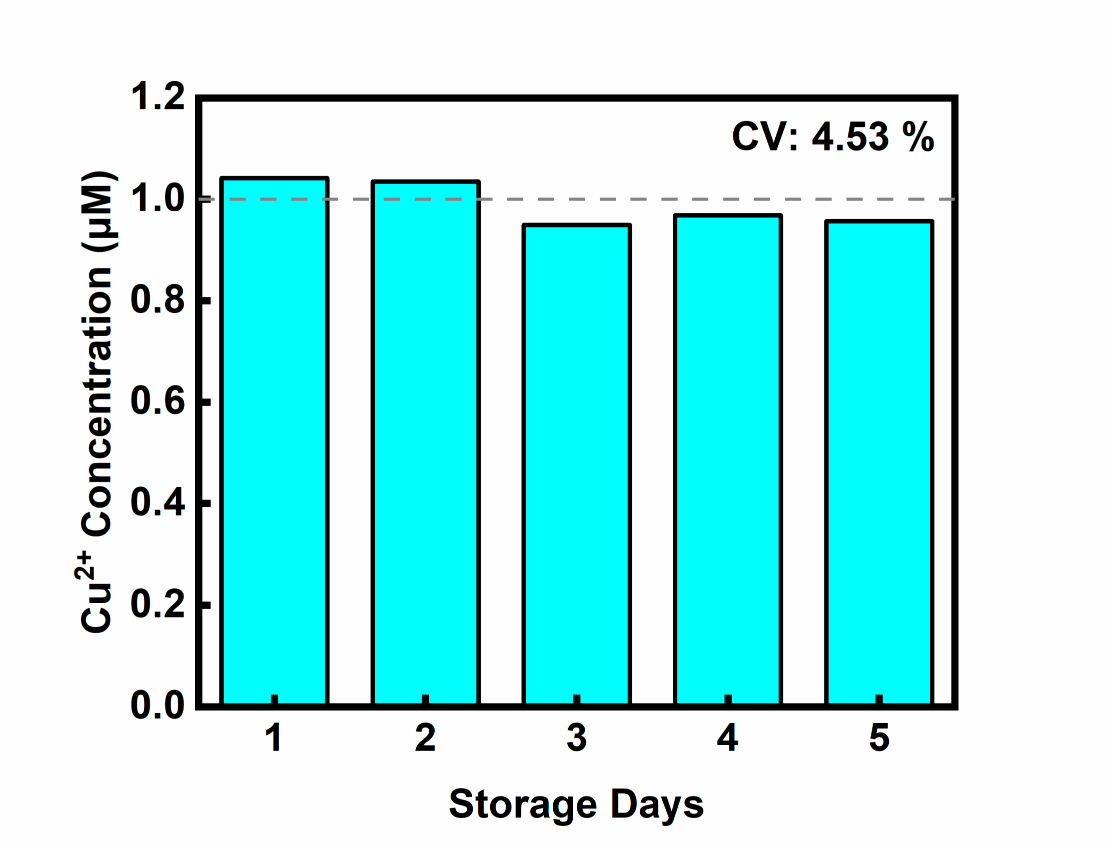

Figure S2. Storage stability of the biological logic gates. The quantitative results of 1 μM Cu^{2+} are detected on the biological logic gates stored in 4 °C for 1–5 days.

Table S1. Comparison of biological logic gates detection capabilities.

Detection Method	Colorimetric	Colorimetric	Fluorescence	Photoelectrochemistry
Cost	Low	Low	High	High
Performance of operations	Simple	Simple	Complicated	Complicated
Liner range	3.91 nM-256 μM	5 nM–1.28 μM	0.01–80 μM	0.1–100 μM
LOD	0.87 nM	0.37 nM	4.2 nM	0.02 μM
References	This work	[1]	[2]	[3]

References

1. Xianyu, Y.L.; Zhu, K.; Chen, W.W.; Wang, X.F.; Zhao, H.M.; Sun, J.S.; Wang, Z.; Jiang, X.Y. Enzymatic Assay for Cu(II) with Horseradish Peroxidase and Its Application in Colorimetric Logic Gate. *Anal. Chem.* **2013**, *85*, 7029–7032.
2. Tong, Y.J.; Yu, L.D.; Wu, L.L.; Cao, S.P.; Guo, Y.L.; Liang, R.P.; Qiu, J.D. Ratiometric Detection of Cu2+ Using a Luminol-Tb-GMP Nanoprobe with High Sensitivity and Selectivity. *ACS Sustain. Chem. Eng.* **2018**, *6*, 9333–9341.
3. Wang, H.; Ye, H.L.; Zhang, B.H.; Zhao, F.Q.; Zeng, B.Z. Electrostatic interaction mechanism based synthesis of a Z-scheme BiOI-CdS photocatalyst for selective and sensitive detection of Cu2+. *J. Mater. Chem. A* **2017**, *5*, 10599–10608.
